# Communication in the Third Dimension: Song Perch Height of Rivals Affects Singing Response in Nightingales

**DOI:** 10.1371/journal.pone.0032194

**Published:** 2012-03-20

**Authors:** Philipp Sprau, Tobias Roth, Marc Naguib, Valentin Amrhein

**Affiliations:** 1 Department of Animal Ecology, Netherlands Institute of Ecology (NIOO-KNAW), Wageningen, The Netherlands; 2 Zoological Institute, University of Basel, Basel, Switzerland; 3 Research Station Petite Camargue Alsacienne, Saint-Louis, France; 4 Behavioural Ecology Group, Department of Animal Sciences, Wageningen University, Wageningen, The Netherlands; Vrije Universiteit, The Netherlands

## Abstract

Many animals use long-range signals to compete over mates and resources. Optimal transmission can be achieved by choosing efficient signals, or by choosing adequate signalling perches and song posts. High signalling perches benefit sound transmission and reception, but may be more risky due to exposure to airborne predators. Perch height could thus reflect male quality, with individuals signalling at higher perches appearing as more threatening to rivals. Using playbacks on nightingales (*Luscinia megarhynchos*), we simulated rivals singing at the same height as residents, or singing three metres higher. Surprisingly, residents increased song output stronger, and, varying with future pairing success, overlapped more songs of the playback when rivals were singing at the same height than when they were singing higher. Other than expected, rivals singing at the same height may thus be experienced as more threatening than rivals singing at higher perches. Our study provides new evidence that territorial animals integrate information on signalling height and thus on vertical cues in their assessment of rivals.

## Introduction

In animal communication, information is often exchanged over long distances. For example, long-range signals such as the song of songbirds can encode information about signaller quality, condition and motivation [1]. However, such information can also be encoded in the spatial behaviour of signallers. For instance, because signals degrade over distance and thus become less detectable [2], [3], distance between sender and receiver can strongly affect the behavioural response of receivers [4]. Also the location of non-moving rivals within [5] and outside [6] the territory boundaries has been shown to influence territory defence behaviour of residents. Moreover, spatial movements of rivals can affect territorial behaviour of resident males [7], [8] and of neighbours [9], thus highlighting the importance of spatial cues in communication.

So far, most studies on communication and territory defence concentrated on effects of spatial behaviour on a horizontal level. However, as animals and particularly birds make use of the three-dimensional space, also the vertical position of signallers is likely to reveal valuable information. Song perch (or song post) height could honestly signal individual quality for several reasons. First, high-quality individuals may be able to defend larger territories, but may need to move upwards to proclaim the ownership of a larger territory over larger distances and to a larger number of rivals, thus potentially creating a link between territory size, perch height and individual quality. Generally, by choosing high perches, signalling males can reduce attenuation of high frequencies caused by foliage [2], [10]. The same foliage effect may also be responsible for enhanced signal reception at high perches [11], [12]. Consequently, exposed perches are considered as being beneficial for long-range communication [13]. Indeed, winter wrens (*Troglodytes troglodytes*) respond to degraded song by choosing higher song perches and thus presumably enhance both their ability to hear distant rivals and the chances to be well heard by the rivals [14].

Second, higher song perches have been shown to lead to higher predation risk by airborne predators, because more exposed signallers are more susceptible to birds of prey [15], [16], [17]. Higher song perches can also increase the costs for thermoregulation due to unfavourable microclimate caused by higher wind speeds and lower temperatures at exposed perches, and therefore be more energy demanding [18]. Song perch height could thus reflect the quality of a signaller, with males singing from high perches being assessed as high quality males, because they can cope with higher energy demands and increased risk of predation. We therefore predict that rivals singing from higher perches are perceived as more threatening during song contests than rivals singing from lower perches.

Here, we examined the effect of rival song perch height on territory defence behaviour in the nightingale (*Luscinia megarhynchos*). Using song playback, we simulated unknown rivals singing from two different heights outside the territories of males. Rivals were simulated as singing either at the same height as the singing resident, or three metres higher than the song perch of the resident. Because songbirds often respond stronger to more aggressive rivals, we predicted resident males to respond stronger to simulated rivals singing from a higher song perch than to those singing at the same level.

## Results

To obtain uncorrelated measures of the subjects' singing responses based on seven different song parameters, we performed a principle component analysis. Two principal components (PC) with Eigenvalues larger than one explained 70% of the total variance in our measured song parameters (Table 1). Temporal song output parameters (i.e. song rate, pause duration, number and duration of interruptions) had high loadings on PC1, whereas all structural song parameters (i.e. percentage of initial whistles and percentage of rapid broadband trills) had high loadings on PC2. Also song length had a high loading on PC2, probably because songs containing trills had longer durations (songs with trills: 3.47±0.06 s (mean ± SE, n = 270 songs pooled from 27 males); songs without trills: 2.92±0.03 s (n = 1077 songs pooled from 27 males). The two principal components were thus taken as reflecting song output (PC1) and structural song parameters (PC2).

**Table 1:** Principle component analysis on seven nightingale song parameters, showing unrotated component loadings.

	PC1	PC2
song rate	**0.47**	−0.26
pause duration	**−0.50**	−0.02
duration of interruptions	**−0.52**	−0.07
number of interruptions	**−0.50**	−0.06
song length	−0.02	**0.65**
songs with trills	0.04	**0.54**
songs with initial whistles	0.08	**0.46**
Eigenvalue	1.84	1.25
variance explained (%)	0.48	0.22

PC1 represents song output parameters, and PC2 represents structural song parameters. Loadings of variables that made an important contribution to the components are indicated in bold. High scores on PC1 indicate high song rates but short durations of pauses and of song interruptions, and low numbers of interruptions; high scores on PC2 are mainly related to long song lengths and high percentage of songs with trills and initial whistles.

**Figure 1:**
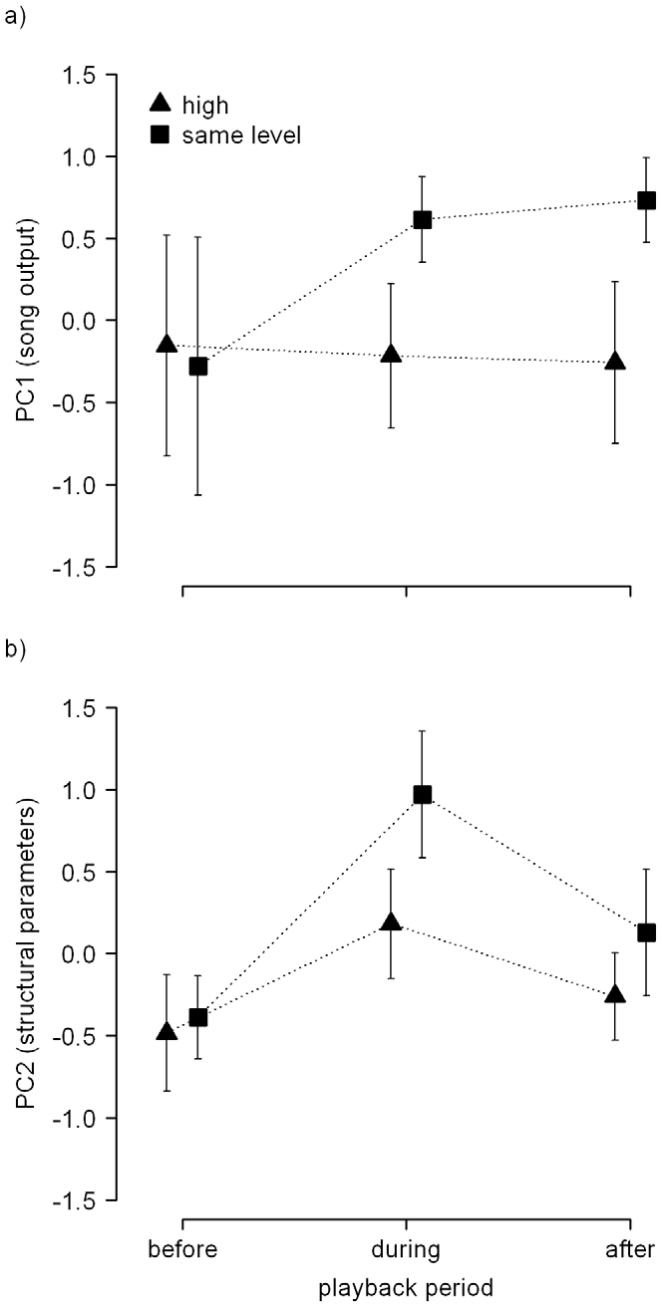
Effect of playback treatment and playback period on mean ± SE song output (a) and on structural song parameters (b) by male nightingales. One group of nocturnally singing males (n = 14) received a playback from the same height as their own song perch (‘same level’), the other group (n = 13) received a playback from 3 metres higher than their own song perch (‘high’). High scores on PC1 (a) indicate high song rates but short pause durations and low numbers and durations of interruptions (see Table 1). High scores on PC2 (b) indicate long song lengths and high percentages of songs with rapid broadband trills and initial whistles.

During playback, other than before playback, males differed in temporal song parameters (PC1) in response to rivals simulated from different heights (Fig. 1a; interaction treatment×playback period: LR = 5.21, *P* = 0.023). Males that were challenged by a simulated rival singing at the same height showed an increase in PC1 during playback, indicating that they increased song rate but decreased pause duration and the use of interruptions. In contrast, when rivals were simulated as singing from high perches, males showed a similar song output during playback as compared to before playback (Fig. 1a). Also after playback, males that received the ‘same level’ playback showed a higher song output compared to before the playback, whereas males that received the ‘high’ playback were singing with a similar song output as before playback (Fig. 1a; treatment×playback period: LR = 5.20, *P* = 0.022).

**Figure 2:**
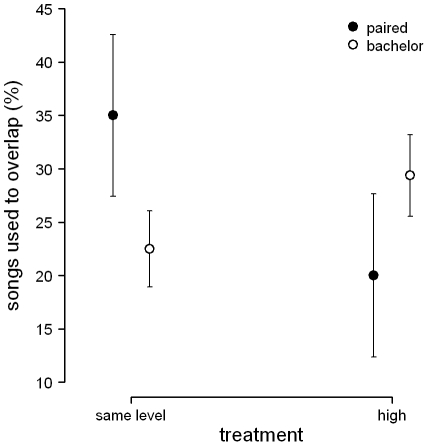
Mean ± SE percentage of their songs that males used to overlap playback songs, in males that later in the season were paired (n = 7), and in males that remained unpaired throughout the breeding season (bachelors; n = 14).

The use of structural song parameters (PC2) during the different playback periods was largely independent of playback treatment (Fig. 1b; during playback versus before playback, treatment×playback period: LR = 0.63, *P* = 0.43; after playback versus before playback, treatment×playback period: LR = 0.09, *P* = 0.76). Also the main effects of treatment were not significant with respect to structural song parameters (Fig. 1b; during playback and before playback: LR = 2.00, *P* = 0.16; after playback and before playback: LR = 1.91, *P* = 0.17). However, males generally showed an increase in PC2 during playback as compared to before playback (Fig. 2b; main effect playback period: LR = 9.66, *P* = 0.002), indicating that in response to both treatments, males increased the use of songs with initial whistles and trills. After playback, they used structural song parameters similarly as before playback (main effect playback period: LR = 2.28, *P* = 0.13).

There was no significant effect of subsequent pairing status (all *P*>0.16) or of subjects' relative song perch height (all *P*>0.16) on temporal and structural song parameters. However, males differed in the percentage of their songs they used to overlap playback songs depending on playback treatment and on subsequent pairing status (treatment×pairing status: LR = 5.61, *P* = 0.018). Subsequently paired males used more songs to overlap the songs of simulated rivals when the rivals were singing from the same height than when rivals were singing from higher perches, whereas bachelors seemed to use more songs to overlap playback songs when the rival was singing from a higher perch (Fig. 2).

## Discussion

Resident male nightingales changed their vocal behaviour stronger in response to playback broadcast from the same height than to playback broadcast from a higher perch. When rivals were simulated as singing from high song perches, males did not significantly change temporal song output parameters over the course of the experiment. In contrast, males increased song output during as well as after playback when songs of rivals were broadcast from the same height: males sang with shorter pauses and with fewer and shorter interruptions, and as a consequence they increased song rate.

Song output has often been interpreted as a trait reflecting male quality [19], [20] as well as the quality of the singer's territory [21], [22]. In vocal interactions, song output is generally considered as a song parameter reflecting the strength of responsiveness to simulated intruders [23], [24] and may be used by eavesdropping females to assess potential mates [25], [26]. Therefore, the increased song output in response to rivals singing from the same level suggests that those rivals were perceived as more threatening than rivals singing from higher perches.

These findings, however, contradict our predictions. We expected that residents would perceive rivals at higher song perches as a greater threat, because high perches may preferentially be used to signal territorial claims to other males over long distances [2]. Moreover, higher song perches could reflect higher quality of a male, because it may honestly signal that it can cope with unfavourable microclimatic conditions [18] and with increased threat from airborne predators [15], [16], [17].

One explanation for the indifferent responses of residents to rivals singing from high song perches could be that those rivals were not perceived as a territorial threat. Instead of signalling territorial claims towards other males, birds singing from high perches may use long-distance advertising predominantly to attract females. In the nightingale, nocturnal song of unpaired males likely serves to attract females that during the period of pair formation prospect the area at night [27]. Moreover, male nightingales are thought to use specific structural song parameters such as whistle songs that begin with a series of repeated notes with a narrow frequency bandwidth to increase detectability for females. Whistle songs transmit over long distances [28] and are preferentially used during nocturnal song [29] and less during male-male interactions [30], suggesting that they indeed serve to attract females over long distances. Thus, nocturnal song sung from high song perches may be interpreted as serving in inter-sexual contexts and may not pose a strong territorial threat to resident males.

Alternatively, unknown rivals singing from high perches may often be non-territorial males prospecting for vacant territories. In nightingales, non-territorial males were shown to prospect several occupied territories before settling in a vacant territory [31] and therefore are likely to leave again after visiting an occupied territory. In a few cases, prospecting males were observed singing for a short time in an occupied territory, from the top of the bushes and above the singing territorial males (VA, HPK & MN, unpublished data). Residents in our study may thus have perceived rivals singing from high perches as non-threatening prospectors and therefore may not have changed song output during playback.

Another possible explanation for our findings could be that predation risk at night is not only caused by aerial predators, but also by ground predators [17]. As a consequence, males singing from lower perches may have been assessed as being exposed to higher risk and therefore as being of higher quality. Further, simulated rivals singing from high perches may have evoked an indifferent response by residents not because they were singing from higher perches, but because they were singing from a different perch height. Thus, complementary studies could investigate whether rivals singing from perches that are lower than the perch of a resident would evoke stronger responses, or whether similarly to high perches, this would lead to indifferent responses by residents.

We also found that subsequently paired males overlapped rivals singing at the same height more often than rivals singing from a higher song perch, whereas bachelors showed a less clear pattern. Previous studies in nightingales showed that during the period of mate attraction, subsequently paired males respond stronger to simulated rivals than do males that stay unpaired throughout the breeding season [32]. As song overlapping is considered an aggressive signal [33], subsequently paired males appear to have perceived the playback broadcast at the same level as more threatening, supporting our results on song output.

Our findings show that different song perch heights of rivals differentially affect the singing response of territorial males, thus highlighting the importance of spatial cues in communication. Earlier studies suggested that spatial movements within [8] as well as across territory boundaries can have strong implications on the behaviour of the resident [7], [34] as well as of neighbours [9]. Other studies showed that also the horizontal location of non-moving rivals within [5] and outside territory boundaries [4], [6] influence territory defence behaviour. Our study provides evidence that in territorial defence, animals also integrate information on song perch height and thus on vertical cues in their assessment of territorial rivals.

## Methods

### Ethics statement

N/A

### Study site and subjects

Nocturnal playback experiments were conducted in the nature reserve Petite Camargue Alsacienne (47°37′20N, 7°32′13E; France). In this area of approximately 18 sqkm, about 200–240 male nightingales occupy territories each year [35]. Most territories are characterized by dense bushes or groves bordering rivers, footpaths, grasslands or open fields, so that territory boundaries usually are well defined by the habitat. During their hourly-long nocturnal singing interactions, males only rarely change songs perches, which allows conducting experiments in standardized contexts, minimising confounding factors such as changes in spatial configurations. Males usually cease nocturnal singing as soon as they get paired, whereas unpaired males continue to sing throughout the breeding season [27], [36]. This allows to distinguish between paired and unpaired males based on standardized census rounds that we made throughout the breeding season to record singing activity of individual males [35].

Playbacks were conducted on 27 territorial males between 29 April and 6 May 2010 at night, between 2300 hours and 0240 hours CEST. All subjects were unpaired during the time of playback. Seven males ceased nocturnal song shortly after the playbacks and were thus considered as ‘paired males’. 14 males continued to sing until late in the breeding season and were thus considered as unpaired ‘bachelors’. For six males we could not unambiguously determine pairing status.

Male nightingales usually sing from the upper half of the shrub layer and from lower parts of the tree layer and occasionally also use exposed song perches ([37], [38]; PS, TR, MN, VA unpublished data). At our study site, the two most abundant nocturnal birds of prey are Long-eared owl (*Asio otus*) and Tawny owl (*Strix aluco*) [VA, TR unpublished data]. In both owl species, birds make up a significant part of diet [39], [40], which is likely to lead to higher predation risk for nightingales singing at higher or more exposed song perches at night.

### Playback Stimuli

To create playback stimuli, we used nocturnal song recordings of 27 different male nightingales made in 2007 or 2008, and each playback stimulus consisted of songs obtained from one male only. Nocturnal song was recorded with a Sony TC-D5M or WM-D6C tape recorder (Sony Ltd., Tokyo, Japan) or a Marantz PMD 660 digital solid state stereo recorder (Marantz Corporation, Kenagawa, Japan) connected to a Sennheiser ME66/K6 microphone (Sennheiser electronic GmbH, Wedemark, Germany). Tape recordings were digitized with Cool Edit 2000 (Syntrillium Software Cooperation, Phoenix, Arizona, USA), and for all recordings we used a sampling frequency of 44.1 kHz with a resolution of 16 bit. Playback stimuli were composed of 20 different songs that were haphazardly chosen from the recordings using the sound analysis software Avisoft SASlab Pro 4.4 (R. Specht, Berlin, Germany). Songs were normalized in peak amplitude using Adobe Audition (Adobe Audition 1.0, Adobe Systems Inc., San Jose, U.S.) and were arranged in a sequence of songs with 3.25 seconds pause duration between the songs, which represents the mean duration of silent intervals in nocturnal song of nightingales (mean ± SD nocturnal pause duration measured for 50 songs from each of 10 males from our study population: 3.25±1.12 seconds). The average duration of the playback stimuli was 297.85±1.84 seconds (mean ± SD), and playback durations did not significantly differ between the two treatments (see below; Welch t-test: *t* = 0.23, *df* = 22.69, *P* = 0.82). Playback stimuli were obtained from recordings made in territories differing from the territories chosen for the experiments, and we also did not use recordings obtained from neighbouring territories. Thus, a subject most likely was unfamiliar with the male whose songs were used for playback. Sound pressure of the stimulus songs was adjusted to 90 dB at 1 m distance, measured with a Voltcraft digital sound level measuring meter SL-300, which is within the range of the sound pressure of singing male nightingales (Brumm 2004).

### Playback Protocol

For the playbacks, we used uncompressed wav files stored on a Foxpro FX5 remote-controlled speaker (Foxpro Inc., United States of America) that was positioned on an extendable metal pole with a maximum length of 6.2 metres. Each of the 27 subjects received one of two non-interactive playback treatments broadcast from an open field bordering the territory. In one treatment group (n = 14 males), playbacks were broadcast from the same height as the subjects' nocturnal song perch (‘same level’). In the other treatment group (n = 13 males), playbacks were broadcast from three metres above the subjects' nocturnal song perch (‘high’).

Territorial males (n = 27) used for the experiment were singing at a mean (± SD) song perch height of 2.64±0.38 m (range: 1.5–3.2 m) above the ground within shrubs. There was no significant difference in song perch height between treatment groups (song perch height of n = 14 subjects during ‘same level’ playback: 2.63±0.48 metres, song perch height of n = 13 subjects during ‘high’ playback: 2.64±0.28 metres; Welch t-test: *t* = 0.08, *df* = 18.98, *P* = 0.94). Maximum height of the trees or shrubs in which the subjects' nocturnal song perches were located ranged from 3 to 20 metres (11.63±5.12 m). The relative height of the territorial males to the canopy measured as the difference between maximum height of the subjects' tree or shrub and their actual song perch height was 8.99±5.08 m (range: 1.0–17.8 m). There was no significant difference in relative song perch height between treatment groups (Welch t-test: *t* = 0.03, *df* = 24.98, *P* = 0.76). Constrained by the length of the extendable metal pole, playbacks were conducted with males that were singing from a maximum song perch height of 3.2 metres, which led to the exclusion of two males because they were singing from higher song perches. These two males were therefore not included in our 27 experimental subjects. The horizontal distance between the loudspeaker and the subjects was 15 metres. The maximum height of the tree or shrub of the subjects' nocturnal song perches were measured on the day following the playback. All distances were measured using a Leica DISTO™ A5 laser distance-metre (Leica Geosystems, Germany).

The vocal behaviour of a subject was recorded during five minutes before the onset of the playback, during the playback, as well as five minutes after the playback, using a Marantz PMD 660 digital solid state stereo recorder (Marantz Corporation, Kenagawa, Japan) connected to two Sennheiser ME66/K6 microphones (Sennheiser electronic GmbH, Wedemark, Germany). On the first channel, we recorded the songs of the subject, and on the second channel, we recorded the songs broadcast by the loudspeaker.

### Response Measures and Statistical Analysis

From the recordings, we measured seven song parameters: (1) song rate (number of songs per minute), (2) song length (s), (3) pause duration (s), (4) duration of interruptions (s), (5) number of interruptions, (6) percentage of songs that were preceded by initial whistles, and (7) percentage of songs that contained rapid broadband trills. Songbirds occasionally interrupt their singing and use these interruptions as a signal in response to rivals [32], [41], [42]. We thus analyzed singing interruptions separately from the regular singing pauses, by defining singing interruptions as silent intervals that were longer than the mean +1 SD of all pauses measured in the 5 minutes before the playback. Accordingly, silent intervals that were longer than 4.80 s were considered as singing interruptions. Rapid broadband trills are often used during close range male-male interactions and therefore are considered as agonistic signals [43]. We defined songs as containing rapid broadband trills when trills in the terminal part of the song had a frequency bandwidth larger than 5000 Hz (measured at −24 dB) and an element repetition rate faster than 8.5 elements per second. Initial whistles were defined as high frequency and low amplitude whistles that are often added to the beginning of nightingale songs particularly in threatening situations [42]. For the period during the playback, as an additional indicator of agonistic behaviour, we also measured the percentage of their songs that subjects used to temporally overlap the non-interactive playback [33], [44].

Data were analysed using R 2.10.1 (R Development Core Team 2009). With the seven song parameters measured during all periods of the playback experiment (i.e. before, during, and after the playback), we performed a principal component analysis using the function ‘prcomp’ in R, to obtain uncorrelated measures of the subjects' singing responses. We used PC-scores as response variables in linear mixed-effects models (LMM) using the lme function in R (package nlme, version 3.1-97). In all LMMs, we included three fixed factors as predictor variables, with two levels each: treatment (‘same level’ or ‘high’), future pairing status (‘paired’ or ‘bachelor’), and playback period (either ‘before’ and ‘during’, or ‘before’ and ‘after’ playback). We ran two different sets of LMMs in which we either tested for changes in response from before to during the playback (i.e. the fixed factor playback period consisted of the levels ‘before’ and ‘during’) or from before to after the playback (i.e. the fixed factor playback period consisted of the levels ‘before’ and ‘after’). We also included all two-way interactions between the three fixed factors. We controlled for the height of the subjects' song perches at the time of playback by including subjects' relative height as a continuous covariate, measured as the difference between maximum height of the subjects' tree or shrub and their actual song perch height. By including relative instead of absolute height of the singing male, we additionally controlled for vegetation structure, which could determine perch height. Because each subject was measured during two playback periods, we used individual subject as a random factor to control for non-independence of data. Non-significant (*P*≥0.05) terms were removed from the models starting with the interactions [45]. The significance of the predictor variables was assessed with likelihood ratio (LR) tests using the maximum likelihood method [46]. For all likelihood ratio tests, the degrees of freedom were *df* = 1.

Song overlapping could only be measured during the actual song playbacks (not before and after the playbacks) and thus was not included into the principal component analysis. We analysed song overlapping with generalized linear models using the glm function in R. The percentage of their songs that subjects used to overlap the playback songs was taken as response variable with a binomial error distribution. Models were selected as described above, with the exception that there were only two fixed factors (treatment and future pairing status).

For all models, we visually checked homogeneity of variance and normality of error using plots of standardized residuals against quantiles from a normal distribution.
